# Peroxisome Proliferator-Activated Receptor α (PPARα) down-regulation in cystic fibrosis lymphocytes

**DOI:** 10.1186/1465-9921-7-104

**Published:** 2006-07-30

**Authors:** Veerle Reynders, Stefan Loitsch, Constanze Steinhauer, Thomas Wagner, Dieter Steinhilber, Joachim Bargon

**Affiliations:** 1 Dept. of Internal Medicine, Division of Pneumology, University Hospital Frankfurt, Germany; 2 Institute of Pharmaceutical Chemistry, University of Frankfurt, Frankfurt am Main, Germany

## Abstract

**Background:**

PPARs exhibit anti-inflammatory capacities and are potential modulators of the inflammatory response. We hypothesized that their expression and/or function may be altered in cystic fibrosis (CF), a disorder characterized by an excessive host inflammatory response.

**Methods:**

PPARα, β and γ mRNA levels were measured in peripheral blood cells of CF patients and healthy subjects via RT-PCR. PPARα protein expression and subcellular localization was determined via western blot and immunofluorescence, respectively. The activity of PPARα was analyzed by gel shift assay.

**Results:**

In lymphocytes, the expression of PPARα mRNA, but not of PPARβ, was reduced (-37%; p < 0.002) in CF patients compared with healthy persons and was therefore further analyzed. A similar reduction of PPARα was observed at protein level (-26%; p < 0.05). The transcription factor was mainly expressed in the cytosol of lymphocytes, with low expression in the nucleus. Moreover, DNA binding activity of the transcription factor was 36% less in lymphocytes of patients (p < 0.01). For PPARα and PPARβ mRNA expression in monocytes and neutrophils, no significant differences were observed between CF patients and healthy persons. In all cells, PPARγ mRNA levels were below the detection limit.

**Conclusion:**

Lymphocytes are important regulators of the inflammatory response by releasing cytokines and antibodies. The diminished lymphocytic expression and activity of PPARα may therefore contribute to the inflammatory processes that are observed in CF.

## Background

Cystic fibrosis (CF) is a common inherited disease caused by mutations in the gene encoding the cystic fibrosis transmembrane conductance regulator (CFTR), which is an epithelial chloride channel. The disorder affects multiple organs and the phenotype is extremely heterogeneous. However, CF morbidity and mortality are mainly due to lung disease, which is characterized by an excessive host inflammatory response. Although CF lung disease is generally considered to be a neutrophil-mediated disorder, recent studies suggest a potent role for lymphocytes in the pathogenesis of the disease [1,2]. In addition, inflammatory markers such as cytokines and eicosanoids are elevated, not only locally, in the airways, but also systemically, thus indicating a more generalized state of inflammation in CF [3-5].

The nuclear factor-κB (NF-κB) and activated protein-1 (AP-1) transcription factors are key players in the inflammatory response by inducing the expression of cytokines, chemokines, cell adhesion molecules and growth factors. The actions of NF-κB and AP-1 can, however, be inhibited by the Peroxisome Proliferator-Activated Receptors α and γ (PPARs), which thereby exert anti-inflammatory properties [6-8]. PPARs are ligand-activated transcription factors belonging to the nuclear hormone receptor super-family. Fatty acids and eicosanoids are natural occurring PPAR ligands [9,10]; fibrates and glitazones are more specific synthetic activators for PPARα and γ, respectively. PPARs regulate gene expression by heterodimerization with the retinoid × receptor (RXR) and subsequent binding to specific DNA sequence elements, termed PPAR response elements (PPRE), in the promoter regions of their target genes [11]. In addition, they can repress gene transcription in a DNA-binding independent manner through inhibition of other signaling pathways by protein-protein interactions and cofactor competition [6,7,12]. At present, three distinct PPAR isoforms have been identified, called α, β and γ. PPARα and γ agonists decrease plasma concentrations of cytokines and acute phase proteins [13-15] and induce anti-atherosclerotic effects [16,17] and are therefore able to influence the immune response. They also seem to play a role in airway inflammation. Similarly, PPARα and γ agonists have been reported to inhibit airway inflammation in a murine model of asthma [18] and a model of airway infection [19] by inhibiting eosinophil, lymphocyte and neutrophil influx into the lung.

Moreover, CF is associated with abnormalities in fatty acid and eicosanoid metabolism. In addition to deficiencies in essential fatty acids in plasma, increased release of arachidonic acid (AA) from the cell membrane and elevated levels of pro-inflammatory eicosanoids in urine, blood and airways have been reported [3,20-24]. Even cell membrane compositions seem to be disturbed with increased levels of AA and decreased levels of docosahexaenoic acid (DHA) [25]. Fatty acids and derivatives can regulate the actions of PPARs and an imbalance may therefore cause inappropriate activation of PPARs.

In conclusion, we hypothesized that the expression of PPARs, transcription factors with anti-inflammatory capacities, is altered in CF. To check our hypothesis, we measured PPARα, β and γ expression in peripheral blood cells, which are important mediators of the inflammatory response through the production and release of cytokines, chemokines, and/or antibodies. We noticed differences for PPARα levels in lymphocytes. Along the same line, an altered PPARα activity was observed in lymphocytes, which confirmed our hypothesis.

## Materials and methods

### Patients

This study was approved by the Ethics Committee of the Frankfurt University Hospital. Patients with cystic fibrosis were between 22 and 43 years old and were all affected by lung disease. They had a stable condition and came for routine check-up. The clinical characteristics of our patients are represented in Table 1. An age-matched, gender-mixed healthy control group was established for all the experiments. Only healthy feeling volunteers, which had not been ill for the past weeks, and which were free from any detectable inflammation, infection or allergic disease were selected for sampling. Due to time, technical and sampling constraints, sample sizes vary between the different experiments.

**Table 1:** Clinical characteristics of cystic fibrosis patients.

**Patient**	**Age (years)**	**Gender**	**Genotype**	**P.a.^1^**	**CRP**	**FEV_1 _% pred^2^**	**FVC % pred^2^**
1	33	F	dF508/R553x	+	0,9	58	91
2	25	M	dF508/dF508	+	0,5	74	90
3	30	M	dF508/dF508	+	0,7	42	72
4	34	M	dF508/?	+	0,3	59	80
5	37	M	dF508/dF508	+	0,6	52	84
6	32	F	dF508/dF508	+	2	60	75
7	26	F	dF508/dF508	+	1,32	86	87
8	28	M	dF508/?	+	0,91	31	48
9	37	M	dF508/dF508	+	< 0,3	30	43
10	32	F	dF508/dF508	+	< 0,3	76	92
11	23	M	dF508/?	+	< 0,3	103	99
12	37	M	dF508/dF508	-	< 0,3	74	101
13	34	F	dF508/dF508	+	0,94	23	59
14	39	M	dF508/dF508	-	< 0,3	60	83
15	25	M	dF508/R553x	+	1,03	85,9	79,7
16	22	F	dF508/N1303	+	0,9	53,6	64,6
17	43	M	dF508/dF508	+	< 0,3	98,8	95,5
18	23	M	dF508/dF508	+	0,8	85,3	84,5
19	39	M	dF508/G542x	+	0,4	61	79
20	40	M	dF508/?	+	0,4	64	80

^1 ^*Pseudomonas aeruginosa *infection

^2 ^Normal: 80–120% of predicted

### Measurement of IL-8 in plasma by ELISA

A commercial ELISA kit was used to measure IL-8 concentrations in plasma (R&D Systems, Germany). The instructions of the manufacturer were followed.

### Measurement of sIL-2R in plasma by ELISA

A commercial ELISA kit was applied to measure soluble IL-2 Receptor levels (R&D Systems, Germany). Prior to use, plasma was diluted 1 to 4. The instructions of the manufacturer were followed.

### Isolation of peripheral lymphocytes and monocytes

To avoid circadian fluctuations of PPARs, blood samples were always taken in the morning. Mononuclear cells were isolated from whole blood by density gradient centrifugation using Lymphoprep (Axis-Shield). After washing with PBS, monocytes were separated from lymphocytes by magnetic sorting (Miltenyi Biotec, Bergisch Gladbach, Germany). Cells were incubated with saturating concentrations of anti-CD14+ monoclonal antibodies conjugated with super paramagnetic particles for 20 min. by 4°C. Subsequently, cells were resolved in PBS (containing 5 mM EDTA and 0.5% BSA) and added on top of a separation column. Unlabeled cells, *i.e*. lymphocytes, were collected through elution from the column. In order to isolate the monocytes, the separation column was detached from the strong magnet and monocytes were eluted. Purity was checked with May-Grünwald Giemsa staining and was ≥ 97%.

### Isolation of peripheral neutrophils

Density centrifugation using Polymorphprep™ solution (Axis Shield, Heidelberg, Germany) enabled us to isolate neutrophils from whole blood. The mononuclear and polymorphonuclear leucocytes were separated into 2 distinct bands, free from red blood cells. Neutrophils were collected, washed with PBS and checked for purity via May-Grünwald-Giemsa staining and had to be > 95%.

### Reverse transcriptase – competitive multiplex PCR/real-time PCR

Total RNA from monocytes, lymphocytes and neutrophils was extracted with RNAzol B™ (Wak-Chemie, Germany) and subjected to oligo(deoxythymidine)-primed first-strand cDNA synthesis using the Superscript II Preamplification System (Invitrogen, Karlsruhe, Germany). The instructions of the manufacturers were followed.

#### Multiplex PCR (see Loitsch *et al*., 1999)[26]

##### Construction of internal standards

The cDNA derived from monocytes and lymphocytes was amplified in the presence of a range of known concentrations of internal standards (competitors). Internal standards for the PPARs and GAPDH were constructed as wild-type fragments containing a deletion of nucleotides: PPARα, β and γ cDNA with a 44, 41 and 106 bp deletion, respectively and GAPDH cDNA with a 55 bp deletion. The shortened fragments were obtained via PCR and the use of following antisense primers: 5'-**ATC ACA GAA GAC AGC ATG GC**C GTT CAG GTC CAA GTT TGC G-3' for PPARα, 5'-**CTG CCA CAA TGT CTC GAT GT**A GGA TGC TGC GGG CCT TCT T-3' for PPARβ and 5'-**TCA GCG GGA AGG ACT TTA TG**C ACT GGA GAT CTC CGC CAA C-3' for PPARγ. The sense primers were the same as those used for the multiplex PCR (see next paragraph). The fragments were ligated in T-vectors (Promega) and the copy number was calculated after spectrophotometric quantification. Then, dilution series (1:3) of the internal standards were established. The internal standards share identical primer recognition sites with the wild-type target.

##### Competitive multiplex Polymerase Chain Reaction

Oligonucleotide primers for PCR were designed according to published sequences: PPARα [GenBank Accession no. Y07619]: sense 5'-TGCAGATCTCAAATCTCTGG-3', antisense 5'-ATCACAGAAGACAGCATGGC-3', amplifying a 374 bp wild-type product; PPARβ [GenBank Accession no. L07592]: sense 5'-TTCCAGAAGTGCCTGGCACT-3', antisense 5'-CTGCCACAATGTCTCGATGT-3'; amplifying a 275 bp wild-type product; PPARγ [GenBank Accession no. D83136]: sense 5'-TCTCTCCGTAATGGAAGACC-3', antisense 5'-TCTTTCCTGTCAAGATCGCC-3', amplifying a 660 bp wild-type product and, GAPDH [GenBank Accession no. M33197]: sense 5'-ATCTTCCAGGAGCGAGATCC-3', antisense 5'-ACCACTGACACGTTGGCAGT-3', amplifying a 502 bp wild-type product.

2–10 μl cDNA was added to a PCR master-mix, which contained all the primers mentioned above. Next, the mix was divided over a series of reaction tubes into which known concentrations of internal standards were spiked. Cycling conditions for PCR were as follows: 94°C for 3 minutes (1 cycle), followed by 40 cycles of 94°C, 58°C, 72°C, each for 45 seconds and a final extension phase at 72°C for 10 minutes (Trio-Thermoblock, Biometra).

The amplification products were separated by agarose gel electrophoresis, stained with ethidium bromide and analyzed by densitometry. Densitometric data were plotted on a log/log scale as a function of internal-standard-derived PCR products and corrected for molar equivalence.

#### Real-time PCR

Neutrophils exhibit low levels of mRNA in general. The classic competitive PCR was not sensitive enough and we had to establish real-time PCR. Real-time PCR was performed by using the ABI prism 7700 sequence detector (Perkin Elmer/Applied Biosystems). Primers and probes were designed using the software program Primer Express (Perkin Elmer/Applied Biosystems). For the measurement of β-actin, a published primers/probe set was applied [27]. The fluorogenic probes contained a reporter dye (FAM) covalently attached at the 5'end and a quencher dye (TAMRA) covalently attached at the 3'end. PPARα [Genbank: NM005036]: sense 5'-CTT CAA CAT GAA CAA GGT CAA AGC-3', antisense 5'-AGC CAT ACA CAG TGT CTC CAT ATC A-3', probe 5'-CGG GTC ATC CTC TCA GGA AAG GCC-3', amplicon length 99 bp; PPARβ [Genbank: L07592]: sense 5'-GGG CAT GTC ACA CAA CGC TAT-3', antisense 5'-GCA TTG TAG ATG TGC TTG GAG AA-3', probe 5'-CTT CTC AGC CTC CGG CAT CCG A-3', amplicon length 147 bp; PPARγ [Genbank: D83233]: sense 5'-GAA ACT TCA AGA GTA CCA AAG TGC AA-3', antisense 5'-AGG CTT ATT GTA GAG CTG AGT CTT CTC-3', probe 5'-CAA AGT GGA GCC TGC ATC TCC ACC TTA TT-3', amplicon length 87 bp; β-actin [Genbank: D28354 and X00351]: sense 5'-AGC CTC GCC TTT GCC GA-3', antisense 5'-CTG GTG CCT GGG GCG-3', probe 5'-CCG CCG CCC GTC CAC ACC CGC C-3', amplicon length 174 bp.

Specific external controls were constructed for all target genes by cloning a partial cDNA fragment (the amplicon of interest obtained by classic PCR amplification) into a pCR^®^2.1 vector (Invitrogen). A standard curve was generated: in each PCR run, 10-fold serial dilutions of the corresponding plasmid clone were included, with known amounts of input copy number. In order to normalize for inefficiencies in cDNA synthesis and RNA input amounts, the mRNA expression of the housekeeping gene β-actin was quantified for each sample. cDNA samples were diluted 10 times prior to PCR amplification. PCR amplifications were performed in a total volume of 25 μl, containing 5 μl cDNA sample, 12.5 μl Taqman Universal PCR Master Mix (Perkin Elmer/Applied Biosystems), 200–800 nM of each primer and 200 nM detection probe (Eurogentec). Each PCR amplification was performed in triplicate, using the following conditions: 2 min. at 50°C and 10 min. at 95°C, followed by a total of 45 two-temperature cycles: 15 s at 94°C and 1 min. at 60°C for PPARs and 67°C for β-actin. PCR data were analyzed through the application of the software 'Sequence Detector 7.1' (Perkin Elmer/Applied Biosystems).

### Western blot analysis for lymphocytes

Equal amounts (80 μg) of total cell proteins were resolved by 10% SDS-PAGE and transferred to a PVDF membrane (Millipore Corporation, Bedford) at 80 V for 1 hour. Membranes were incubated with mouse PPARα monoclonal antibodies (1:2000 dilution) (clone B11.80A, generous gift from Dr. Winegar, Glaxo Smith Kline) at 4°C overnight. Protein levels were normalized using a mouse monoclonal antibody against β-actin (1:10.000 dilution) (Sigma). Proteins were subsequently detected through the use of horseradish peroxidase-conjugated secondary antibodies and the chemiluminescence system ECL (Amersham Pharmacia Biotech, Buckinghamshire, UK). After scanning (DocuGel V-System, Scananalytics), band intensities were analyzed using the software package Zero-D-Scan™ (Scananalytics).

### Immunofluorescence assay

Cytospin glass slides were prepared by centrifugation of 10^5 ^lymphocytes using a cytospin centrifuge (Cytospin 4, Thermo Shandon). After cells were fixed in ice-cold methanol and blocked with a solution of 2% BSA in PBS overnight at 4°C, they were permeabilized with Perm/Wash buffer (BD Biosciences Pharmingen) and then incubated for 2 hours with monoclonal PPARα antibody, diluted 1:10 in Perm/Wash/2%BSA buffer (clone Pα B32.51 kindly provided by Dr. Winegar, Glaxo Smith Kline [28]). After washing, the second antibody (cy3 labeled goat-anti-mouse, Caltag) was added in a 1:250 dilution in Perm/Wash/2%BSA buffer for 30 min. Following washing and air-drying, the cells were embedded in Aquatex (Merck) and evaluated by immunofluorescence microscopy.

### Gel shift assay

Nuclear proteins from lymphocytes were prepared as described by Dignam and coworkers [29]. A gel shift kit for PPARα was obtained from Panomics, Inc. and the instructions of the manufacturer were followed. Equal amounts of nuclear protein extracts (10 μg as determined by Bradford assay) were incubated for 30 min. with biotin-labeled oligonucleotide probe, which corresponds to the PPAR binding site, and then subjected to non-denaturing PAGE. Afterwards, proteins were blotted on a PallBiodyneB^® ^(PALL Corporation) membrane and bands were visualized after exposure to Hyperfilm™ECL (Amersham Biosciences, UK). Subsequently, equal loading was checked via Coomassie Blue staining of the membrane. Band intensities were analyzed using the software package Zero-D-Scan™ (Scananalytics).

### Statistical analysis

Results are expressed as mean ± SE. Statistical comparisons were made using the unpaired Student's t-test (Sigma-plot). A value of p < 0.05 was considered significant.

## Results

### Interleukin-8 levels in plasma

**Figure 1:**
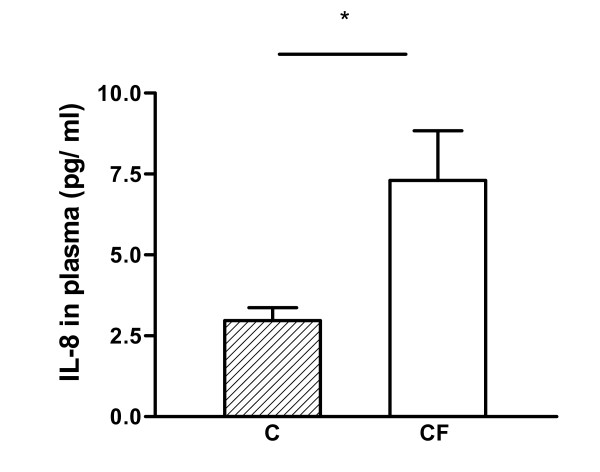
IL-8 levels in plasma of CF patients and healthy persons measured by ELISA. IL-8 levels are significantly higher in CF patients (n = 15) than in control persons (n = 11). Results are shown as mean ± standard error. * Significantly different (p < 0.03).

IL-8 in plasma was measured via ELISA to demonstrate that the patients in this study exhibit the typical elevated systemic cytokine levels [30]. As expected, IL-8 levels were significantly higher in CF patients compared with control persons (7.3 pg/ml vs 2.9 pg/ml, respectively; p < 0.03) (Fig. 1). We can therefore assume that the inflammation cascade is not restricted to the airways, but is also found systemically.

### PPAR mRNA expression in peripheral blood cells

In order to check for differences in the expression of PPARs between CF patients and healthy persons, we started screening at mRNA level. All data were normalized to the expression levels of the housekeeping genes GAPDH or β-actin, which were equally expressed in samples of CF patients and control persons.

#### Monocytes and lymphocytes

**Figure 2:**
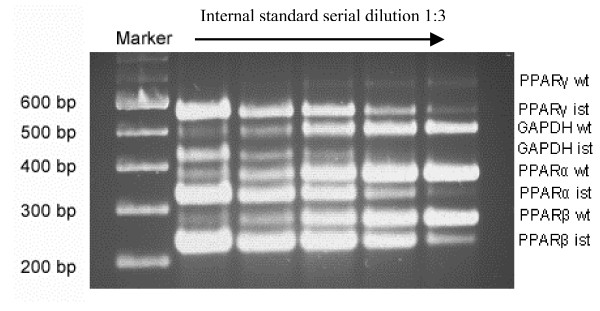
RT-competitive multiplex PCR for PPARα, β, and γ and GAPDH in human peripheral blood lymphocytes. Picture of ethidium bromid stained agarose gel after electrophoresis of the amplified products. wt = wild-type amplicon, ist = internal standard amplicon.

Competitive multiplex PCR products were loaded on an agarose gel, electrophorised and stained with ethidium bromide (see fig. 2). Bands were scanned and analyzed with the software package Zero-D-Scan™ (Scananalytics).

##### PPARα

**Figure 3:**
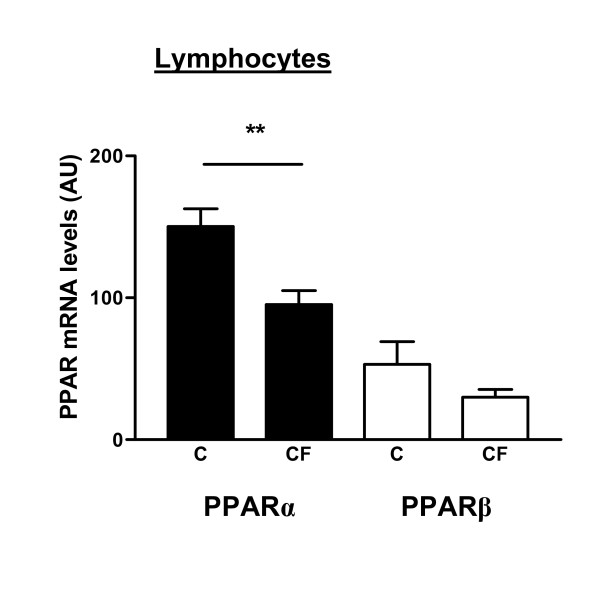
Human peripheral blood lymphocytes from CF patients (CF, n = 15; M(ale)/F(emale): 9/6) and healthy subjects (C, n = 11; M/F: 6/5) were subjected to RT-competitive multiplex PCR and densitometry in order to measure PPARα and PPARβ mRNA expression levels. Data are normalized to GAPDH expression levels. Values are represented as means ± standard error. PPARα expression levels were 37% lower in CF patients compared to control persons. ** Significantly different (p < 0.002).

**Figure 4:**
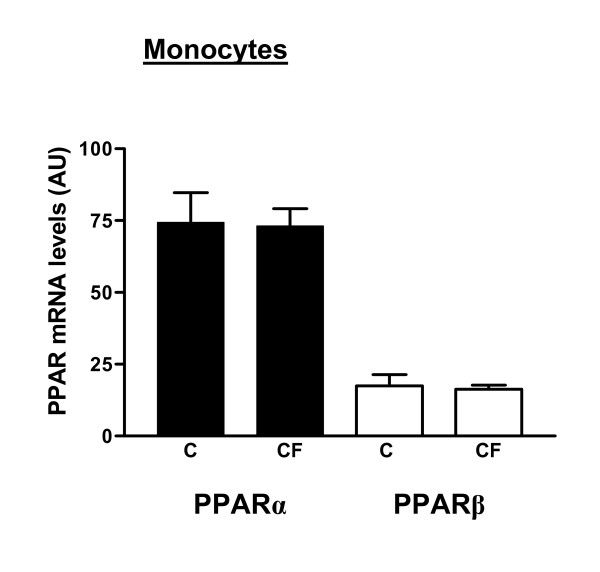
PPARα and PPARβ mRNA expression levels were measured in human peripheral blood monocytes from CF patients (CF, n = 19; M/F: 13/6) and healthy subjects (C, n = 10; M/F: 6/4) via RT-competitive multiplex PCR and densitometry. Data are normalized to GAPDH mRNA expression levels. Values are means and standard error. Both PPAR levels were similar in the two groups.

PPARα mRNA levels were significantly lower (-37%, p < 0.002) in lymphocytes of CF patients compared with control persons (Fig. 3). In monocytes, no differences were observed in the expression of PPARα between the healthy subjects and the CF patients (Fig. 4).

##### PPARβ

For both lymphocytes and monocytes, no statistical differences in the mRNA expression of PPARβ were detected between CF patients and healthy persons (Fig. 3 and 4).

##### PPARγ

PPARγ mRNA was detected in a few samples of monocytes and lymphocytes, but was not quantifiable due to the extremely low expression levels.

#### Neutrophils

Neutrophils are considered end-cells as DNA and most, but not all, mRNA and protein synthesis, cease once the myeloid cells are mature enough to enter the blood. For that reason, mRNA levels were rather low in neutrophils and PPAR mRNA was difficult to quantify via the classic competitive multiplex PCR. We therefore developed real-time PCR, a highly sensitive and accurate method.

##### PPARα

**Figure 5:**
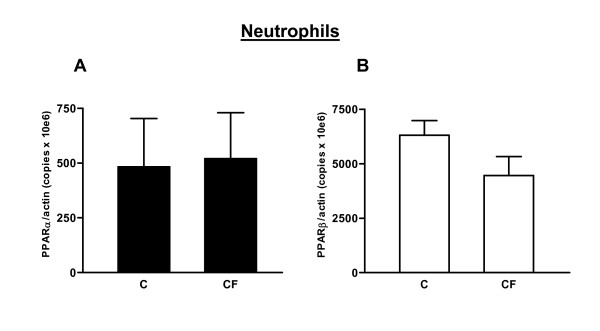
PPAR mRNA expression in freshly isolated human neutrophils was determined by real-time PCR for CF patients (n = 12; M/F: 7/5) and control persons (n = 12; M/F: 6/6). Data are presented as the mean PPAR mRNA level relative to the β-actin mRNA expression [(number of PPAR copies/number of β-actin copies) × 10^6^] and the standard error. Each measurement was performed in triple. (A) PPARα mRNA expression. (B) PPARβ mRNA expression. No differences were seen between the two groups for both PPARα and PPARβ mRNA levels.

PPARα mRNA levels were equal in neutrophils of CF patients and healthy persons (Fig. 5A)

##### PPARβ

Idem, PPARβ mRNA levels were similar in both groups (Fig. 5B).

##### PPARγ

PPARγ mRNA was detectable, but the low expression levels did not allow quantification.

### PPARα protein levels in peripheral blood lymphocytes measured via western blotting

**Figure 6:**
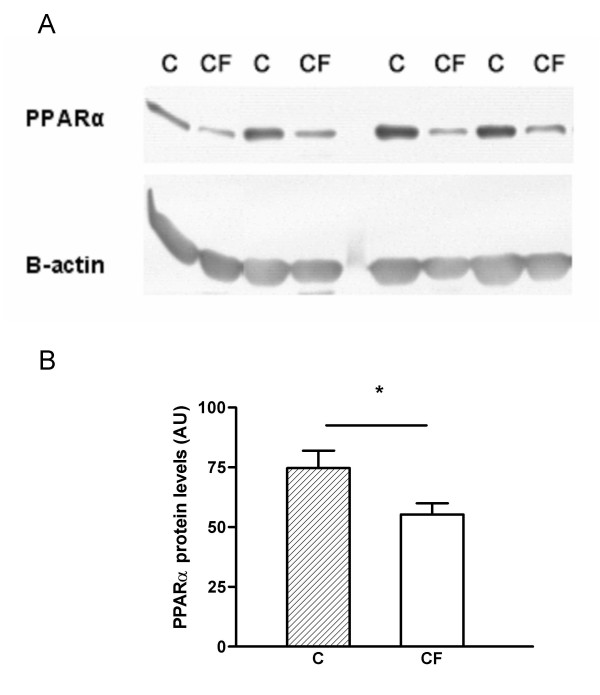
Western blot analysis of total protein extracts derived from human peripheral blood lymphocytes from control persons (C, n = 10; M/F: 4/6) and CF patients (CF, n = 11; M/F: 6/5). (A) A single band was detected at 60 kDa for PPARα. β-actin protein expression was measured for normalization. (B) Analysis of band intensities revealed that PPARα protein levels are down-regulated (-26%) in CF patients compared to healthy subjects. Densitometry data are expressed as means ± standard error. * Significantly different (p < 0.05).

mRNA analysis revealed less expression of PPARα in lymphocytes of CF patients compared with healthy persons. On the basis of this finding we further examined the expression of the receptor at protein level via western blotting. A single band for PPARα was observed around 60 kDA (Fig. 6A). Analysis of the band intensities demonstrated that protein levels of PPARα are significantly lower (-26%, p < 0.05) in lymphocytes of CF patients compared with control subjects (Fig. 6B). β-actin was measured for normalization.

### Localization of PPARα in human peripheral blood lymphocytes

**Figure 7:**
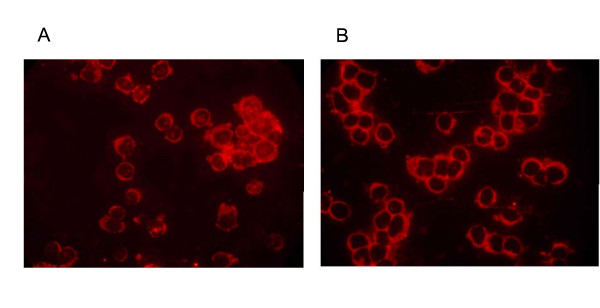
The subcellular localization of PPARα protein in freshly isolated human peripheral blood lymphocytes was determined via immunofluorescence assay (C: n = 5 and CF: n = 5). Microscopy analysis revealed that the transcription factor is mainly situated in the small cytoplasmic area. (A) Representative immunofluorescence picture of lymphocytes derived from healthy control blood and (B) from a CF patient.

In order to identify the subcellular localization of PPARα within peripheral blood lymphocytes, an immunofluorescence assay was developed. As shown in Fig. 7A and 7B, the highest concentration of the protein is observed in the cytosol, whereas the nucleus contains only trace amounts of the transcription factor. In the context of our study, the technique was not found appropriate for quantifying PPARα protein levels by means of measuring the fluorescence intensity. Activity was therefore measured via gel shift assay.

### Activity of the PPARα transcription factor

**Figure 8:**
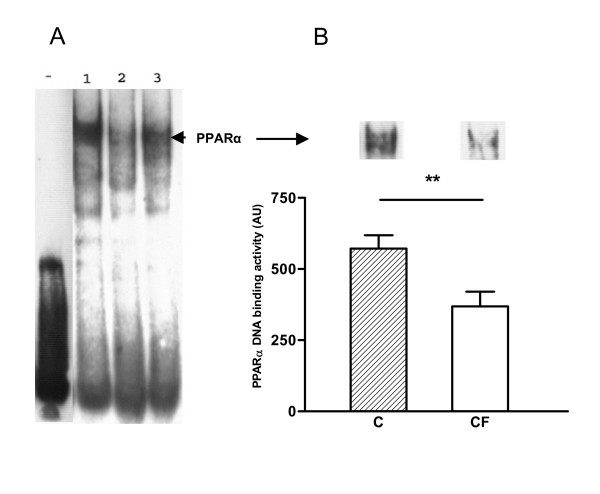
Differential PPARα binding to PPRE in peripheral lymphocytes. PPARα DNA binding was analyzed via gel shift assay in peripheral lymphocytes of CF patients (CF, n = 11; M/F: 6/5) and healthy subjects (C, n = 11; M/F: 6/5). The DNA binding element was biotin-labeled. (A) A representative gel shift. Lane (-) represents the biotin-labeled DNA binding element, without the addition of nuclear extract. Lane 1: Lymphocytic control sample. Lane 2: Specific cold oligonucleotide binding competition assay: a 60-fold excess of cold synthetic PPRE was added. Lane 3: Unspecific cold oligonucleotide binding competition assay. A 60-fold excess of unspecific synthetic oligonucleotide was used. (B) Densitometry data derived from the gel shift assays are expressed as means and standard error. These data show that PPARα DNA binding activity of CF patients is reduced by 36% compared to healthy persons. ** Significantly different (p < 0.01). On top: representative bands from a control person and a patient.

Since PPARα expression is lower in lymphocytes of CF persons, it was deemed useful to check for the activity of the transcription factor, which was determined via gel shift assay (Fig. 8). To this end, a commercially available kit for PPARα was used (Panomics). The DNA-binding element (PPRE) was not radioactive-, but biotin-labeled. Equal amounts of nuclear extracts were loaded. The measurement of band intensities showed that PPARα DNA binding activity was 36% less in lymphocytes of CF patients, compared with control subjects (p < 0.01) (Fig. 8B). In order to evaluate the binding specificity, competition analysis was performed by adding 60-fold cold specific (PPRE) and unspecific oligonucleotide (see fig. 8A: lane 2 and 3, respectively). The upper band fainted strongly by adding cold PPRE, but remained unaltered after adding cold unspecific oligonucleotide. Equal loading of nuclear extracts was verified via Coomassie Blue staining of the membrane.

### sIL-2 R levels in plasma

**Figure 9:**
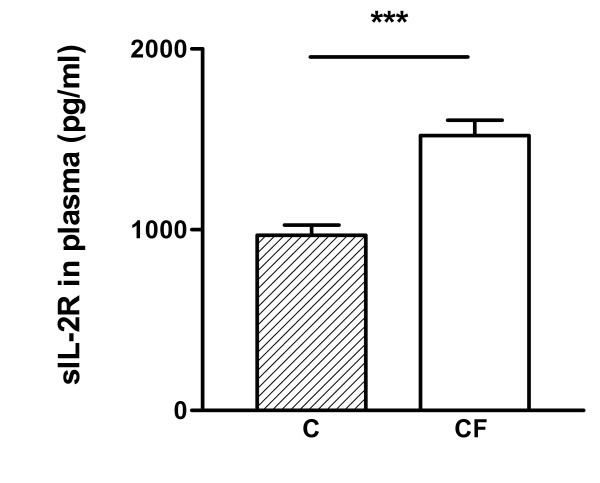
sIL-2R levels were measured in plasma of CF (n = 19) and control persons (n = 18). sIL-2R levels are significantly higher in CF patients than in control persons. Data are represented as mean and standard error. *** p < 0.0001.

Soluble IL-2 receptor (sIL-2R), a well-known marker for T-lymphocyte activation, was measured in plasma of stable CF patients and control persons via ELISA (Fig. 9). Normal values for sIL-2R levels in plasma are around 1020 pg/ml. Statistical analysis revealed that CF patients exhibit significantly higher levels of sIL-2R in plasma than healthy persons (CF: 1521 ± 84.15 pg/ml vs C: 970 ± 56.44 pg/ml). These data indicate that peripheral T-lymphocytes of CF patients are more activated than lymphocytes of healthy subjects.

## Discussion

The mechanisms behind the disturbed immune response in CF are still largely unknown and require further research. The aim of our study was to measure the expression of the PPAR transcription factors in patients with cystic fibrosis and healthy subjects. Because of their known regulatory functions in inflammatory processes, we hypothesized that their expression and/or function may be altered in cystic fibrosis, a disorder characterized by an excessive host inflammatory response.

Our study confirmed that systemic inflammation was present in our CF patients on the basis of the observed increased levels of plasma IL-8. Our data revealed that both PPARα mRNA and protein levels were significantly lower in peripheral lymphocytes of CF patients than in healthy control persons. Immunofluorescence experiments demonstrated that just a small fraction of PPARα resides in the nucleus, whereas the cytosol contains the larger part of the transcription factor. This was observed for both groups. Differences in activity were demonstrated via gel shift assay, i.e. a significant reduction of PPARα DNA binding activity in lymphocytes of CF persons compared with healthy subjects. Finally, increased levels of soluble IL-2 Receptor in plasma suggest that peripheral lymphocytes are activated in cystic fibrosis.

Most CF patients become chronically infected with specific bacterial pathogens, such as *Pseudomonas aeruginosa*, which cause a destructive inflammatory response in the lung. However, several studies provide evidence that inflammation can occur prior to infection and that CF lungs are primed for inflammation [30-32]. Nevertheless, the inflammatory processes are not restricted to the respiratory tract as shown by the elevated levels of pro-inflammatory markers in the blood circuit of CF patients [4,30,33]. Our study also demonstrated elevated levels of IL-8 in plasma of CF patients. Therefore, monocytes, lymphocytes and neutrophils were studied, as they are important mediators of the inflammatory response, *i.a*. through the release of cytokines, chemokines, and through the production of antibodies.

Our study revealed that PPARα and PPARβ are abundantly expressed in freshly isolated monocytes and lymphocytes at mRNA level, whereas little or no PPARγ was detected. Both PPARα and PPARβ mRNA could be measured via real-time PCR in neutrophils; PPARγ mRNA on the other hand was not quantifiable. Statistical analysis showed that PPARα mRNA, but not PPARβ mRNA, is significantly less expressed (-37%) in lymphocytes of CF patients compared with control persons. The same difference could be detected at protein level via western blotting. The expression of PPARα and β mRNA in monocytes and neutrophils was not significantly different in patients and healthy persons. These data are supported by several studies. First, there is evidence that PPARα mRNA and protein expression are directly regulated by its own ligands [34,35]. Fatty acids and eicosanoids, which are natural PPAR activators, are found in disturbed levels in CF and may therefore cause a diminished expression of PPARα. Second, PPARα expression within T-lymphocytes is rapidly down-regulated following cellular activation [36]. Our present study demonstrated increased plasma soluble interleukin-2 receptor (sIL-2 R) concentrations in CF patients, which is in line with the findings of other research groups [37,38]. sIL-2 R is a generally accepted marker for T-lymphocyte activation [39]. Therefore, T-lymphocytes appear to be in some sort of activated state in CF patients, which may be responsible for the decreased PPARα levels. The mechanism responsible for this down-regulation has not yet been elucidated. Third, the pro-inflammatory cytokines IL-6, TNF-α and IL-1 have been demonstrated to cause a reduction in the expression of PPARα [40,41]. CF patients exhibit increased levels of IL-2, TNF-α, IL-6 and IL-8 in sputum and serum [4,5,32,37]. However, this can not be the major explanation for the decreased PPARα levels in lymphocytes, as the expression of the transcription factor was unaltered in monocytes and neutrophils. And finally, an interesting abstract by Andersson and team reported that a CF tracheal epithelial cell line expressed less PPARα protein than a normal tracheal epithelial cell line, which is comparable with our data [42]. The same research team found decreased PPARγ levels in tissues specifically regulated by CFTR in a CF mice model [43] and their data suggest that CFTR may play a role in PPAR expression. A functional CFTR is also expressed in lymphocytes of healthy humans. Consequently, a defect CFTR in CF lymphocytes could result in altered PPAR expression. In addition, research has shown that PPAR expression may differ significantly in target organs where inflammation occurs. For example, a recent study reported that induction of PPARα is lacking in the liver of CF mice compared to wild type animals following colitis induced bile duct injury [44].

Following our findings that PPARα expression is down-regulated in CF lymphocytes, the question arose whether the activity of the transcription factor was also altered. Our immunofluorescence experiments revealed that for both groups, the transcription factor is primarily located in the cytosolic compartment and only a small fraction resides in the nucleus. A similar cellular distribution was reported in human macrophages [45] and in mice lymphocytes [36]. This meant that gel shift analysis had to be applied to measure possible differences in the activity of PPARα. The gel shifts indeed showed that PPARα DNA binding activity was 36% lower in lymphocytes of CF patients compared with control persons. A decreased DNA binding activity of PPARγ was also seen in tissues of CFTR knock-out mice. Treatment of these mice with rosiglitazone, a PPARγ agonist, restored DNA binding [43].

**Figure 10:**
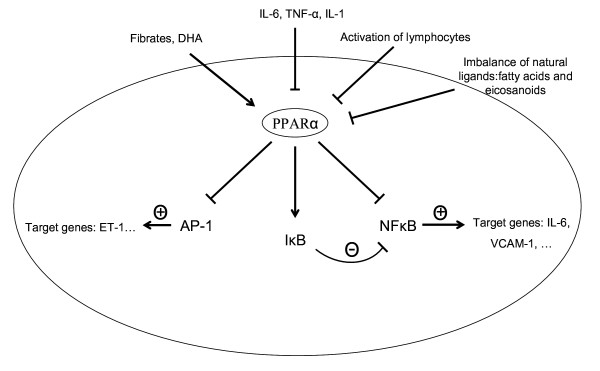
Summarizing picture: PPARα in lymphocytes. PPARα inhibits the actions of the pro-inflammatory transcription factors AP-1 and NFκB through protein-protein interactions and by up-regulating the expression of IκB. The nuclear hormone receptor is inhibited by specific cytokines and by lymphocyte activation. In addition, we suggest that the imbalance of natural ligands in CF leads to deficiencies in PPARα activation. Therefore, lymphocytes may be turned into cells that promote inflammation in CF. We hypothesize that the addition of synthetic or natural ligands, such as fibrates and DHA respectively, may restore the activity and expression of PPARα, resulting in a more balanced lymphocytic immune response.

PPARα was the first isotype recognized for its *in vivo *role in inflammatory processes. Inflammation induced by leukotriene B4, a PPARα ligand, has been reported to be prolonged in PPARα knock-out mice, suggesting an anti-inflammatory role for PPARα [46]. Ligand-induced activation of PPARα in lymphocytes antagonized DNA binding activity of NF-κB and decreased IL-2 and TNF-α production [36,47], inhibited IFN-γ secretion but promoted IL-4 secretion and production [47,48]. These data indicate that PPARα may have a significant influence on the lymphocytic immune response. Consequently, a decrease in PPARα expression and function may contribute to the excessive host inflammatory response. Our data suggest that administration of ligands, such as the natural DHA or synthetic fibrates, may serve as a therapy to help reduce the inflammatory processes in CF by upregulating the activity of PPARα (see fig. 10). Further studies need to be carried out to test this hypothesis.

CF lung disease is well-known as a neutrophil-mediated disease. However, as pointed out by Moss, the behavior and biological function of lymphocytes is also altered in CF. Lymphocytes are important immune cells because they determine the specificity of the immune response. Quantitative analysis of inflammatory cells in CF lung tissues revealed a lymphocyte-dominated immune response in the CF bronchial wall, beneath the surface epithelium [1]. These lymphocytes may release cytokines, such as IL-17, that may attract neutrophils into the airways [49,50]. CF peripheral lymphocytes also exhibit an altered pattern in cytokine-release and production after stimulation [51-53], which could indicate an impairment of the immune response at the systemic level. Moreover, CF lymphocytes are characterized by a specific incapacity to respond to *P. aeruginosa *antigens [54]. Consequently, this defect could contribute to the inability to eradicate lung infection and inflammation due to *P. aeruginosa*. Summarized, the function of lymphocytes is altered in CF and they are therefore an interesting target to be studied.

In conclusion, our study revealed that both the expression and activity of PPARα, a transcription factor with anti-inflammatory capacities, is down-regulated in peripheral lymphocytes of CF patients, which may render lymphocytes into cells that promote the inflammatory response and consequently lead to increased inflammation. In addition, the natural activators of PPARα are known to be present in disturbed proportions in CF and may therefore cause an improper activation of PPARα. We therefore hypothesize that the expression and activity of PPARα may be up-regulated via the administration of natural or synthetic agonists which eventually may lead to a diminished immune response.

## Abbreviations

AA: Arachidonic acid

AP-1: Activator protein-1

CF: Cystic fibrosis

CFTR: Cystic fibrosis transmembrane conductance regulator

DHA: Docosahexaenoic acid

NF-κB: Nuclear factor-κB

PPAR: Peroxisome Proliferator-Activated Receptor

PPRE: PPAR response element

## Competing interests

The author(s) declare that they have no competing interests.

## Authors' contributions

VR carried out the experiments, wrote the manuscript and participated in the study design. SL designed the multiplex competitive PCR for PPARs, participated in the study design and helped evaluating the results and techniques. CS provided technical assistance. TW and DS provided the work with critical comments. JB participated in the coordination of the project and corrected the article.
